# How to improve crop pathogen resistance with epigenetics

**DOI:** 10.1186/s42483-025-00393-7

**Published:** 2026-01-27

**Authors:** Litsa Ampntelnour, Amelia Burton, Vardis Ntoukakis

**Affiliations:** School of Life Sciences, University of Warwick, Coventry, CV4 7AL UK

**Keywords:** DNA methylation, Histone acetylation, Chromatin remodelers, Plant immunity mechanisms, Plant-pathogen interactions

## Abstract

Crop losses due to pathogens are a critical global challenge threatening food security and agricultural sustainability. Recent advances in plant science have highlighted the role of epigenetic regulation in plant immunity. This review examines current insights into how plants utilize epigenetic modifications, focusing on histone modifications, DNA methylation changes, and chromatin remodelling, to modulate defence gene expression and adapt to biotic stress. We explore how histone modifications influence both basal and induced immune responses through key enzymes and we examine the dual roles of DNA methylation in transcriptional activation and repression. Additionally, the review discusses ATP-dependent chromatin remodelling complexes and non-coding RNA to highlight their contribution in fine tuning plant immunity. Finally, we discuss emerging technologies involved in epigenome editing and identify the knowledge gaps that must be addressed before translating our findings into making resilient agricultural systems.

**Supplementary Information:**

The online version contains supplementary material available at 10.1186/s42483-025-00393-7.

## Background

Plant pathogens are a significant threat to global agriculture, causing substantial annual losses in crop yield and quality, which directly impact food security and economic stability. The Food and Agriculture Organization (FAO) estimates that pathogens are responsible for annual crop losses worth $220 billion and are worsened by post-harvest losses (FAO, 2021). Moreover, pressures like the growing global population and the intensifying effects of climate change make the development of disease-resistant crops an essential strategy for ensuring agricultural sustainability and resilience (Savary et al. 2019; Ristaino et al. 2021). Traditional breeding approaches, despite their challenges, have achieved considerable success in enhancing plant immunity (Nelson et al. 2018; Li et al. 2021), but are yet to take advantage of epigenetics.

Epigenetic mechanisms, such as histone modifications, DNA methylation, chromatin remodelling, and non-coding RNAs processing, allow plants to dynamically adjust gene expression without altering the underlying DNA sequence (Hannan Parker et al. 2022; Wu & Fan., 2025). These processes could influence key pathways involved in pathogen defence and enhance biotic stress resilience of plants. During the past decade, considerable efforts have been made to reveal the mechanisms underlying plant epigenetic mechanisms and how they can enhance plant immunity, which in combination with their conservation across plant lineages offer a promising avenue for crop improvement.

This review aims to explore the link between plant epigenetic regulation and the plant’s challenge to reprogram transcription during infection by fungal pathogens, bacteria, and oomycetes. We also summarized the novel contributions of both forward and reverse genetics studies in plants connecting histone acetylation, DNA methylation, and chromatin remodelling to plant immunity. However, due to space limitation, epigenetic responses to viral infections are not discussed in this review. By summarizing the advancements in this field, we hope to highlight the importance of integrating epigenetic insights into modern breeding strategies to develop resilient crops for the future.

## Introduction

### Plant immunity: an overview of the main mechanisms

Plants counteract pathogens by a tightly integrated immune system, consisting of both innate immunity which is genetically encoded and adaptive responses influenced by environmental conditions. Unlike animals, where innate and adaptive immunity are distinct, these systems in plants are interconnected and operate through shared molecular pathways (Mauch-Mani et al. 2017, Yuan et al., 2021). The innate immune system is activated by signals that include pathogen associated molecular patterns (PAMPs), the molecules derived from invading pathogens (Bigeard et al. 2015; Boutrot and Zipfel., 2017), or damage associated molecular patterns (DAMPs), the molecules produced as a consequence of host cellular damage (Li et al. 2020a, b). These signals are recognized by pattern recognition receptors (PRRs) located on the surface of plant cells and stimulate a core defence mechanism defined as PAMP triggered immunity (PTI) activating downstream responses, such as activation of mitogen-activated protein kinase (MAPK), reactive oxygen species (ROS) burst, and phytohormone accumulation, including salicylic acid (SA), jasmonic acid (JA), and ethylene (ET) (Macho & Zipfel., 2014). As a counterattack, some pathogens secrete effector molecules to suppress PTI and this leads to either effector-triggered susceptibility (ETS) or in the case of recognition from the plant’s intracellular receptors (NLRs)—encoded by R genes—it leads to effector-triggered immunity (ETI) (Jones& Dangl., 2006; Keller et al. 2016; Wersch et al. 2020). Unlike PTI, ETI induces stronger and longer-lasting responses and signals for hypersensitive response (HR) which is associated with localised cell death that could restrict most pathogen proliferation (Cui et al. 2015). Both receptor classes mentioned are highly interconnected, with activation of one pathway capable of influencing the other (Wu & Fan., 2025, Yuan et al., 2021). While a few receptors can function independently of helper proteins, most PRRs and NLRs operate by interacting with coreceptors, forming intricate networks. In many cases, they function synergistically: NLR-mediated responses often rely on PRR activity, certain PRR-triggered outputs depend on NLR components, and salicylic acid (SA) perception is essential for full activation of both PRR- and NLR-mediated immunity (Liu et al. 2020, Ngou et al., 2021, Yuan et al., 2021, Pruitt et al. 2021).

In contrast to the genetically fixed nature of innate immunity, plants also exhibit a more flexible, environmentally responsive defence strategy known as induced resistance (IR). This form of phenotypic plasticity allows plants to modify their defence responses based on specific external signals (De Kesel et al. 2021). Induced resistance could be triggered by microbe perception, plant volatiles or endogenous stress signals (Conrath et al. 2006; Heil and Ton 2008). Common examples of IR responses are systemic acquired resistance (SAR) predominantly mediated by SA (Conrath et al. 2015) and induced systemic resistance (ISR) after colonisation by plant-beneficial microbes which primarily relies on JA/ET pathways (Pieterse et al. 2014). Since IR enhances existing innate immune responses, the pathways and mechanisms controlling IR and innate immunity overlap. Τhese multi-layered immune strategies highlight the complexity of plant defence and emphasize the importance of regulatory mechanisms, such as epigenetic control, in fine-tuning immune responses.

### Epigenetic regulations orchestrate immunity responses to pathogens

Following recognition of invading pathogens, plants are faced with a challenge to regulate activation, silencing, and duration of immunity-related gene expression. This is where epigenetic regulation becomes essential, enabling plants to modulate gene expression not only at transcriptional level but through reversible and heritable modifications at the chromatin level that directly influence accessibility of defence genes.

The basic unit of chromatin is the nucleosome, which is composed by approximately 146 bp of DNA wrapped around a histone octamer, comprising of two molecules of each of the histones H2A, H2B, H3, and H4 (Luger et al., 1997). Nucleosomes are joined by internucleosomal DNA forming a “beads-on-a-string” structure (Luger et al. 2012; Rutowicz et al. 2019). Open chromatin regions, or euchromatin, are typically associated with transcriptionally active genes, whereas condensed heterochromatin is generally transcriptionally silent and enriched in repetitive sequences and transposable elements (Vergara and Gutierrez 2017). Epigenetic mechanisms including histone modifications, DNA methylation, and chromatin remodelling can alter the degree of chromatin accessibility and, consequently, gene expression under pathogen stress. These pathways do not act in isolation, rather they operate in parallel to form a regulatory network needed for robust immune responses.

## The role of histone modifications in regulating plant immunity

**Fig.1:**
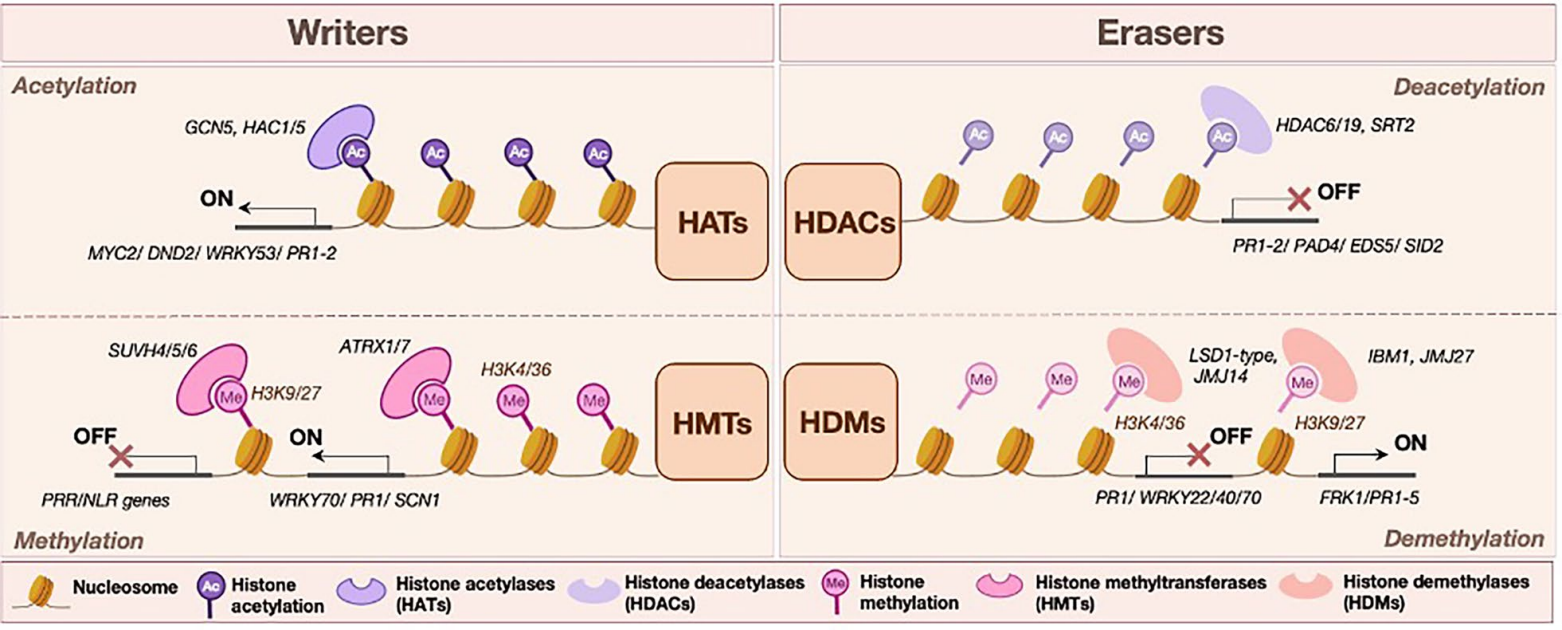
Regulation of immunity related transcription through histone modifications. 'Writers' such as histone acetyltransferases (HATs) and histone methyltransferases (HMTs) add acetyl (Ac) or methyl (Me) groups to histones, leading to activation (ON) or repression (OFF) of immunity genes expression depending on the type of mark. 'Erasers' like histone deacetylases (HDACs) and histone demethylases (HDMs) remove these marks, reversing the immunity outcome

Histone modifications play a pivotal role in regulating plant immunity, with key enzymes such as histone acetyltransferases (HATs), histone deacetylases (HDACs), histone methyltransferases (HMTs), and histone demethylases (HDMs) orchestrating these processes. Together, these enzymes establish a dynamic epigenetic landscape that enables plants to adapt to pathogen challenges, as illustrated in Fig. 1.

### Histone acetylation and deacetylation: balancing immunity

In general, histone acetylation, catalysed by HATs generally functions to activate gene expression by relaxing chromatin structure, while deacetylation by HDACs results in gene repression through chromatin compaction. For instance, HAC1 (Histone acetyltransferase 1) in *Arabidopsis thaliana* has been identified as a positive regulator of PTI by promoting the expression of early defence genes like *WRKY53* (WRKY DNA—binding protein 53), *FRK1* (flg22—induced receptor-like kinase 1), and *NHL10* (NDR1/HIN1-like 10) with *hac1* mutants exhibiting susceptibility against *Pseudomonas syringae* pv. *tomato* DC3000 in *A. thaliana* (Singh et al., 2014a; Jin et al. 2018). Similarly, *GCN5* (General control nonderepressible 5) has emerged as a central transcriptional activator in both *A. thaliana* and soybean, driving the expression of defence-related genes, such as *WRKY33*, *MYC2* (Myelocymatosis 2), and members of the *NAC* (NAM, ATAF1/2, and CUC2) family, against the oomycete *Phytophthora sojae* (Kong et al. 2017). In addition to its activating role, *GCN5* functions as a repressor of salicylic acid (SA) accumulation, through the modulation of H3K14 (Lysine-9 of H14) acetylation levels on its targets and balances defence responses with other physiological processes (Kim et al. 2020).

In tomato, H3K9 (Lysine-9 of H3) acetylation correlates with increased resistance to *Botrytis cinerea* through activation of oxylipin biosynthesis genes and the transcription factor *WRKY75* (Crespo-Salvador et al. 2020). In wheat, *TaHAG1*-mediated acetylation of *PAD4* (Phytoalexin deficient 4) leads to SA accumulation and ROS production, both of which are critical in conferring resistance against *Blumeria graminis* f. sp. *tritici* (Song et al., 2022). Likewise, nitric oxide treatment on *A. thaliana* has been linked with hyperacetylation of immunity genes and accumulation of SA (Mengel et al. 2017). Beyond these canonical HATs, the elongator complexes *AtELP2* and *AtELP3* contribute to defence by modifying chromatin during transcriptional elongation. Mutants lacking the elongator components display delayed expression of defence genes and reduced resistance against *B. cinerea* and *A. brassicicola*, indicating their dual role in basal and ETI pathways (Defraia et al., 2010, 2013). *AtELP2* also directly influences JA/ET pathway induction, with loss-of-function mutants showing severe immune impairment against the same pathogens (Wang et al. 2013). In cassava, the HAM1 acetyltransferase, along with the chaperone *DNAJA2*, activates SA biosynthesis and defence gene expression, conferring enhanced resistance to *Xanthomonas axonopodis* pv. *manihotis* upon overexpression (Zhao et al. 2023).

In comparison to HATs, HDACs often act as negative regulators of plant immunity by repressing the transcription of defence-associated genes. This suppressive role is evident in *A. thaliana*, where *HDAC6* targets genes such *as PR1* (Pathogenesis related protein 1), *PR2, CBP60g* (Calmodulin-like gene), and *SARD1* (Systemic acquired resistance deficient 1), which are central to SA biosynthesis and signalling (Wang et al. 2017a, b). *HDAC9*, another member of the RPD3 family, represses stress tolerance responses by post-translationally inhibiting WRKY53 transcription activity (Zheng et al. 2020). Interestingly, *HDAC19* appears to play a more nuanced role: while it represses *PR1* and *PR2* genes, its overexpression enhances resistance to *Altenaria brassicicola*, suggesting that it likely acts as a fine-tuner of SA pathway amplitude (Choi et al. 2012). In rice, *HDAC701* functions as a broad-spectrum repressor of immune genes, and its knockout leads to increased resistance to *Ustilaginoidea virens*, the causal agent of rice false smut (Chen et al. 2022). Similarly, *OsSRT2*, which is targeted by the fungal effector Uv1809 during *U. virens* infection, suppresses host immunity by deacetylating key defence loci; however, *ossrt2* mutants display enhanced resistance without growth penalties, making it a promising target for crop engineering (Chen et al., 2024). Other sirtuin-type HDACs like *SRT2* in *A. thaliana* also contribute to immune repression by downregulating *PAD4, EDS5* (Enhanced disease susceptibility 5)*, SID2* (Salicylic acid induction-deficient 2)*,* and *PR1* (Wang et al. 2010). Additional repressors include *HD2B*, which modulates PTI via suppression of flg22-responsive genes (Latrasse et al. 2017) and *HDT701*, where silenced transgenic lines were resistant to *Magnaporthe oryzae* as well as *Xanthomonas oryzae* pv*. oryzae (Xoo)* (Ding et al., 2012). Zhi et al., further demonstrated that the same deacetylase in wheat led to enhanced powdery mildew resistance in knocked down mutants (Zhi et al., 2020).

Beyond immediate immune responses, histone acetylation has also been implicated in transgenerational resistance, where the next generation of plants show enhanced defense responses due to stress or pathogen exposure in the parental generation. One proposed mechanism is that pathogen challenge induces stable chromatin modifications that are maintained through gametogenesis and transmitted to offspring, thereby “priming” their immune system. Recent work demonstrated that the biocontrol strain *Bacillus velezensis* K165 (previously known as *Paenibacillus alvei*), not only conferred immediate protection to *Verticillium dahliae* in *A. thaliana* but also established inheritable immunity in the progeny of treated plants through HATs mediated priming of defence-related genes, including *CAD3*, which is involved in lignin biosynthesis (Gkizi et al. 2021).

While these examples highlight the role of histone acetylation in plant immunity, how this modification interacts with other histone marks to modulate defence responses remains poorly understood. A notable exception is the work of Jaskiewicz et al., which showed that local infection with *P. syringae* pv. *maculicola* led to systemic chromatin changes, including H3K9 & H4K5/8/12 acetylation and trimethylation of H3K4 (lysine-4 of H3) on promoters of *WRKY29/6/53*. These modifications were detected not only on the site of infection but also in distal uninfected leaves, indicating a SAR effect (Jaskiewicz et al. 2011).

### Histone methylation and demethylation regulators

In comparison to histone acetylation, methylation is more complex due to the fact that distinct methylation patterns are linked to gene activation or repression. Generally, H3K4me3 and H3K36me2/3 (lysine-3 of H36) are associated with transcriptionally active regions, whereas H3K9me2 and H3K27me3 (lysine-3 of H27) are associated with silenced regions. This makes demethylation by HDMs context-dependent, as it can either promote gene activation or lead to transcriptional silencing, depending on the specific mark being removed.

Several enzymes target H3K4 methylation and are implicated in plant immunity regulation. In *A. thaliana*, *ATRX1* (Arabidopsis trithorax 1) promotes resistance by depositing H3K4me3 and activating *WRK70* and *PR1* in response to *Pst* DC3000 (Alvarez-Venegas et al. 2007)*.* A closely related protein, ATRX7, interacts with *MOS9* to boost the expression of NLR’s like *SNC1* and *RPP4*, which get activated in ETI responses, against *Hyaloperonospora arabidopsis* (Xia et al. 2013). In contrast, demethylases such as LSD1-like proteins (LDL1, LDL2, and LDL4) can remove H3K4 marks. LDL1 and LDL2 work redundantly to repress *PR1* and key transcription factors like *WRKY22/40/70* while their double mutant show altered immunity against *Pst* DC3000 (Noh et al. 2021). LDL4 encodes an LSD1-like protein and its loss impairs the plant’s ability to mount an effective SAR response against *Pst* DC3000 (Singh et al. 2013, 2014b). It was lately shown that LDL4 acts as a positive regulator of plant defence against the necrotrophic fungi *B. cinerea* and *Alternaria alternata*. More importantly, *ldl4* mutants are partially defective in JA signalling, but hyperactive in ethylene signalling (Singh et al. 2019).

Similarly, JMJ14 suppresses defence gene expression by erasing pathogen-induced H3K4 marks, fine-tuning the SA signalling pathway (Li et al. 2020a, b). This was also observed in rice, where JMJ704 reduces H3K4 levels to silence negative regulators of defence, such as *NRR* and *WRKY63*, enhancing resistance against *Xoo* (Hou et al., 2015). Set domain group 25 (SDG25) enhances resistance to both *B. cinerea* and *A. brassisicola* and is an example of an H3K4 methyltransferase which modulates defence responses by influencing both histone methylation and H2B ubiquination (Lee et al. 2016).

Most of the studies on H3K36 methylation show that it is regulated by SDG8 methyltransferase in *A. thaliana*. SDG8 promotes the expression of a wide range of defence-related genes (*MYC2*, *PDF1.2a*, *PR1/2*, and *NPR1)*, and wax and carotenoid biosynthesis genes like *CCR2/CCR3*. Knockout mutants of SDG8 show compromised resistance to fungal pathogens *B. cinerea*, and *A. brassicicola* and bacterial pathogen *Pst* DC3000 and *Pst* DC3000 *hrpA* strains which show faster HR (Berr et al. 2010; Palma et al. 2010; De-La-Pena et al. 2012). Notably, SDG8 represents a clear example of conserved function across species, as its orthologs in tomato, SDG33 and SDG34, also mediate the deposition of H3K36 marks. This functional conservation is further supported by the increased susceptibility of SDG33/34 mutants to *B. cinerea* infection (Bvindi et al. 2022).

Modifications at H3K9, typically linked to gene repression, are also supported by some studies. SUVH4/KRYPTONITE, along with SUVH5 and SUVH6, silence immune receptors under normal conditions to prevent unnecessary activation (Cambiagno et al. 2021). However, IBM1 and JMJ27 counteract this repression during infection by removing H3K9 marks, activating *FRK1* (a PTI marker) and a broad set of pathogenesis related genes (*PR1–PR5*) respectively (Chan & Zimmerli et al., 2019; Dutta et al. 2017). Downstream responses of H3K9 methylations marks can also consist of a mixture of PTI and ETI responses like the case of SRT1 in rice, where H3K27 marks trigger ROS bursts, MAPK signalling, HR, DNA fragmentation, and programmed cell death (PCD) (Huang et al. 2007).

H3K27 methylation further layers this response, where a complex like PRC2 adds repressive H3K27 marks to control genes involved in PCD (Dvorak Tomastikova et al., 2021), while REF6 removes them, priming *PRR* and NLR genes for quick response (Cambiagno et al. 2021). LHP1, which also binds to H3K27, represses *MYC2* and its loss leads to reduced pathogen resistance (Ramirez-Prado et al., 2019). While most H3K27 methylation work is focused on *A. thaliana*, Li et al., showed that in rice, JMJ705 enhances resistance against *Xoo* when overexpressed, further supporting the conserved role of these marks across species (Li et al. 2013).

### Other histone modifications

In addition to the histone acetylation and methylation pathways, other distinct histone-related mechanisms can alter plant immune responses (Table 1). For instance, *HUB1/HUB2* in *A. thaliana* and tomato activate immunity through H2B monoubiquination, where mutations lead to increased susceptibility to a number of pathogens including *B. cinerea, A. brassisicola,* and *V. dahliae* (Dhawan et al. 2009; Hu et al. 2014; Zhang et al. 2015). Additionally, linker histone H1 variants in *A. thaliana* influence DNA methylation and histone acetylation, with mutations improving resistance to pathogens like *Pst* DC3000 and *B. cinerea* (Sheikh et al. 2023).

**Table 1:** Histone modifications involved in plant responses to pathogens

HMTs & HDMs involved in plant responses to pathogens				
Modifiers	Plant species/Pathogen	Modification site	Influence on immunity	References
*ATX1—*(*ARABIDOPSIS HOMOLOG OF TRITHORAX*)	*A.* *thaliana/Pst* DC3000	H3K4 deposition	Activation	Alvarez—Venegas et al., (2007)
*ATRX7—*(*ATX1 RELATED 7*)	*A.* *thaliana/H. arabidopsis*	H3K4 deposition	Activation	Xia et al. (2013)
*FLD—(FLOWERING LOCUS D)/LDL4*	*A.* *thaliana/Pst* DC3000	H3K4 removal	Activation	Singh et al. (2013), (2014b)
*LDL1—(LSD1 LIKE 1) & LDL2*	*A.* *thaliana/Pst* DC3000	H3K4 removal	Repression	Noh et al. (2021)
*FLD—(FLOWERING LOCUS D)/LDL4*	*B. cinerea & A. alternata*	H3K4 removal	Activation	Singh et al. (2019)
*JMJ14—(JUMONJI14)*	*A.* *thaliana/Pst* DC3000	H3K4 removal	Activation	Li et al. (2020)a, b
*OMJ704—(JUMONJI704)*	*Oryza sativa/X.o* pv. *oryzae*	H3K4 removal	Repression	Hou et al., (2015)
*AtSDG25—*(*SET DOMAIN GROUP 25*)	*A.* *thaliana/* *A. brassisicola &* *B. cinerea*	H3K4 deposition	Activation	Lee et al. (2016)
*AtSUVH4—(SU(VAR)3–9 HOMOLOG) /KRYPTONITE (KYP)*	*A. thaliana/Pst* DC3000	H3K9 deposition	Repression	Cambiagno et al. (2021)
*AtSUVH5/6*	*A.* *thaliana/Pst* DC3000	H3K9 deposition	Repression	Cambiagno et al. (2021)
*AtIBM1—(INCREASE IN BONSAI METHYLATION 1)/JMJ25*	*A.* *thaliana/Pst* DC3000	H3K9 removal	Activation	Chan and Zimmerli (2019)
*AtJMJ27—(JUMONJI27)*	*A.* *thaliana/Pst* DC3000	H3K9	Activation	Dutta et al. (2017)
*AtLHP1—(LIKE HETEROCHROMATIN PROTEIN1)*	*A. thaliana/Pst* DC3000	H3K27 deposition	Activation	Ramirez- Prado et al., (2019)
*PRC2—(POLYCOMB REPRESSIVE COMLEX 2)*	*A.* *thaliana/Pst* DC3000	H3K27 deposition	Repression	Dvorak Tomastikova et al., (2021)
*REF6—(RELATIVE EARLY FLOWERING 6) /JMJ12 (JUMONJI12)*	*A.* *thaliana/Pst* DC3000	H3K27 removal	Activation	Cambiagno et al. (2021)
*JMJ705—(JUMONJI705)*	*Oryza sativa/X. o *pv. *oryzae*	H3K27	Activation	Li et al. (2013), De La Pena et al., (2012)
*SDG8—(SET DOMAIN GROUP 8)*	*A.* *thaliana/Pst* DC3000	H3K36 deposition	Activation	Lee et al. (2016), Palma et al. (2010)
	*A.* *thaliana/A. brassisicola & B. cinerea*	H3K36 deposition	Activation	Berr et al. (2010)
*SDG33 & SDG34—(SDG8-orthologues)*	*S.* *lycopersicum/B. cinerea*	H3K36 deposition	Activation	Bvindi et al. (2022)

Modifiers	Plant species/Pathogen	Influence on immunity		References
*HATs & HDACs involved in plant responses to pathogens*				
*HAC1—(HISTONE* *ACETYLTRANSFERASE 1)*	*A.* *thaliana/Pst* DC3000	Activation		Singh et al., (2014a); Jin et al. (2018)
H3K9 acetylation	*S. lycopersicum/B. cinerea*	Activation		Crespo-Salvador et al. (2020)
*GCN5—(GENERAL CONTROL NONDEREPRESSIBLE 5)*	*A.* *thaliana/Pst* DC3000	Activation		Kim et al. (2020)
HAG1—*(HISTONE* *ACETYLTRANSFERASE 1)*	*T. aestivum/B. graminis* f. sp. *tritici*	Activation		Song et al., (2022)
ELP2 & ELP3- (ELONGATOR COMPLEX)	*A.* *thaliana/B. cinerea—A.brassicicola*	Activation		Defraia et al., (2010) & Defraia et al., (2013)
ELP2—(ELONGATOR COMPLEX)	*A.* *thaliana/Pst* DC3000	Activation		Wang et al (2013)
HAM1 & DNAJA2 (DNAJ HEAT SHOCK PROTEIN FAMILY)	*Manihot esculenta/X. axonopodis* pv. *manihotis*	Activation		Zhao et al. (2023)
HDAC6 (HISTONE DEACETYLASE 6)	*A. thaliana/Pst* DC3000	Repression		Wang et al. (2017)a, b
HDAC9 (HISTONE DEACETYLASE 9)	*A.* *thaliana/Pst* DC3000	Repression		Zheng et al. (2020)
HDAC19 (HISTONE DEACETYLASE 19)	*A. thaliana/A. brassisicola*	Repression		Choi et al. (2012)
HDAC701 (HISTONE DEACETYLASE 701)	O. *sativa/U. virens*	Repression		Chen et al. (2022)
SRT2 (targeted by effector Uv1809)	*O. sativa/U. virens*	Repression		Chen et al. (2024)
SRT2 (SIRTUIN LIKE HISTONE DEACETYLASE)	*A.* *thaliana/Pst* DC3000	Repression		Wang et al. (2010)
HD2B variant	*A.* *thaliana/Pst* DC3000	Repression		Latrasse et al. (2017)
HDT701—(HISTONE DEACETYLASE 701))	*T. aestivum/B. graminis* f.sp. *tritici*	Repression		Zhi et al., (2020)
HDT701—HISTONE DEACETYLASE 701)	*O. sativa /M. oryzae—X. oryzae* pv. *oryzae*	Repression		Ding et al. (2012)
HDAC705—(RPD3-like HDAC)	*O. sativa/U. virens*	Repression		Chen et al., (2024)

*Other histone modifications*				
HUB1/HUB2—(HISTONE MONOUBIQUINATION 1/2)	*A.* *thaliana/B. cinerea—A.brassisicola*	Activation	Dhawan et al. (2009)	
	*A. thaliana/V. dahliae*	Activation	Hu et al. (2014)	
	*S. lycopersicum/B. cinerea*	Activation	Zhang et al. (2015)	
H1 variant—linker histones	*A.* *thaliana/Pst* DC3000—*B. cinerea*	Activation	Sheikh et al. (2023)	

## DNA methylation in plant-pathogen interactions

**Fig. 2:**
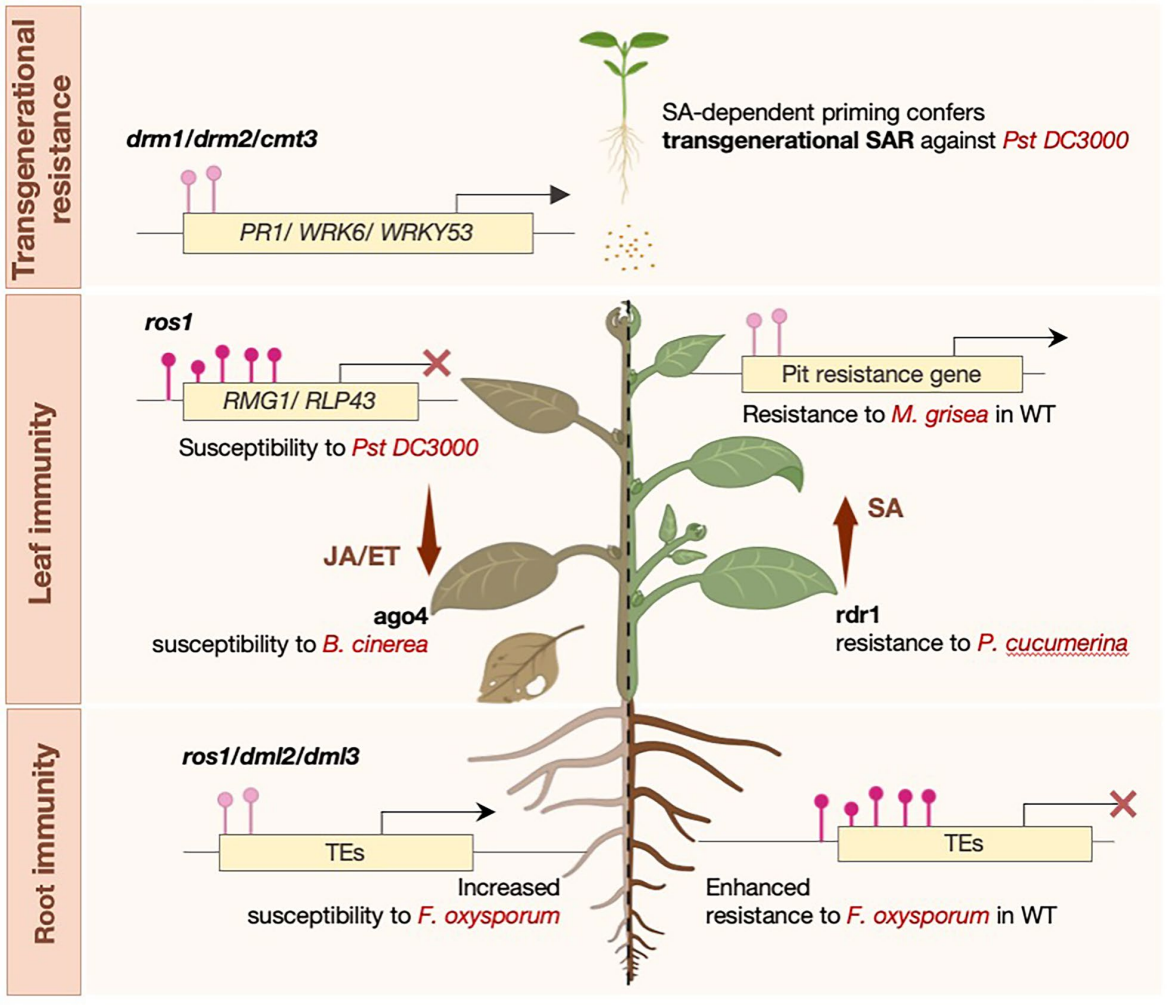
DNA methylation shapes plant immunity in a tissue-specific and transgenerational manner. This figure illustrates examples where methylation changes lead to disease susceptibility (left) and cases where they promote resistance (right). Additionally, certain DNA methylation marks can confer SA-dependent immune priming that is heritable and enhances resistance in the next generation. Pink marks above DNA regions indicate whether hypomethylation or hypermethylation is associated with each response

Compared with histone modifications, most studies of DNA methylation in plant immunity have centered on bacterial pathogens, particularly *Pst* DC3000, where methylation impacts promoter regions, gene bodies, and transposable elements, as well as SAR and transgenerational priming. DNA methylation refers to the addition of a methyl group to cytosine residues in DNA, and for plants it primarily occurs at CpG sites (Lang et al. 2017; Zabet et al. 2017). Broadly, DNA methylation has two functions in plant-pathogen interactions: it affects pathogen adaptation and virulence while also modifying host defensive mechanisms (Lang et al. 2017; Saze et al. 2003; Zabet et al. 2017) as shown in Fig. 2.

In the host, pathogen-induced methylation changes at promoter regions often regulate immunity genes and are usually induced upon pathogen infection. One of the first examples are the multiple cytosine hypomethylations induced by *Pst* DC3000 (Pavet et al. 2006). Specifically, hypomethylations could target promoters of a core immunity gene, such as the promoter of *NPR1* in *A. thaliana* where its expression is compromised upon *Pst* DC3000 infection (Pavet et al. 2006) or target a large group of gene promoters, like flg22-induced global hypomethylation that enhances PTI-mediated responses against the same pathogen (Yu et al. 2013). Similarly, hypermethylation can also have a negative influence on immunity like in *A. thaliana* mutant *ros1* that exhibit increased methylation levels at the promoters of *RMG1* and *RLP3* which reduces SA-mediated resistance to *Pst* DC3000 (Yu et al. 2013 & Halter et al., 2021).

On the contrary, gene body methylation has a more nuanced, context-dependent effect on gene expression as its impact varies on whether the methylation occurs in CG, CHG or CHH sequence. Several studies on crops such as cassava, rice, common bean, and tomato have provided valuable insights into the association of gene body methylation and the regulation of NLRs and other immunity-related genes, although the functional relevance during pathogen attack remains unclear (Li et al. 2012; Wang et al. 2015; Kim et al. 2015; Wang et al. 2017a, b).

Transposable elements (TEs) also play a critical role in shaping immune responses as their mobility within immunity-related genes increases under stress. In rice, TEs affect WRKY45 function through TE-derived siRNAs that modify *ST1* methylation and the plant resistance to *Xoo* (Zhang et al., s). In *A. thaliana*, pericentromeric TEs near PRR/NLR genes compromise defence when regulated via the RdDM pathway (Cambiagno et al. 2018).

Conversely, many studies link DNA methylation changes to induced resistance. Hypomethylation in mutants, such as *cmt3*, *drd1,* and *nrpe1*, display enhanced SA-mediated resistance to *H. arabidopsis* and bacterial pathogens, while hypermethylation in mutants like *ros1* resulted in increased susceptibility to *Pst* DC3000 (López et al., 2011; Cambiagno et al. 2021). Luna et al., showed that methylation is also linked to transgenerational priming of resistance in the case of *drm1/drm2/cmt3 (ddc)* triple mutant, where *Pst* DC3000 infection leads to DNA hypomethylation of defence genes and consequent SAR which is extended across generations (Luna et al. 2012). Similarly, in rice, hypomethylation in the promoter of resistance gene *Xa21G* confers transgenerational resistance to *Xoo,* the causal agent of rice bacterial blight disease (Akimoto et al. 2007). Moreover, Wibowo et al., highlighted the importance of organ-specific epigenetic memory in shaping immune responses in plants (Wibowo et al. 2018). Arabidopsis plants regenerated asexually from root tissue exhibited enhanced resistance to *Pst* DC3000 compared to those regenerated from leaf tissue. This differential immune phenotype was linked to the partial retention of root-specific DNA methylation patterns, suggesting that tissue-of-origin epigenetic marks can influence defence outcomes even in genetically identical plants (Wibowo et al. 2018).

Interestingly, identical DNA methylation patterns can have opposite effects on immunity depending on the genomic area in which they occur. In the *ddc* mutant, hypomethylation enhances resistance to *Pst* DC3000, supporting its role in activating immune responses. However, the same hypomethylation pattern leads to increased susceptibility to *Agrobacterium tumefaciens*, as shown by the enhanced crown gall tumor formation in both *ddc* and *ago4* mutants (Gohlke et al. 2013). Further studies showed that, increased susceptibility to *Pst* DC3000 was observed in plants with the *AGO4* mutant alleles *ago4-1* and *ago4-2*, which suggests that *AGO4* has a unique function in plant disease resistance compared with other RdDM factors (Agorio and Vera., 2007; López et al. 2011).

Fungal infections often modify the methylation status of promoters and TEs to alter gene transcription (Le et al. 2014; Deng et al. 2017). For instance, *A. thaliana* triple DNA demethylase mutant *rdd* (*ros1/dml2/dml3*) exhibit increased susceptibility to *Fusarium oxysporum* due to enrichment of stress-response gene promoters with short sequence TEs (Le et al. 2014). Similarly, *A. thaliana* mutants deficient in siRNA synthesis (*rdr6* and *dcl2/3/4*) or methylation maintenance *(ddc* mutants) demonstrate high susceptibility to a range of necrotrophs like *B. cinerea, Plectosphaerella cucumerina,* and *A. brassicicola* (Cai et al. 2018; Luna et al. 2012; López et al., 2011).

In contrast, downregulation of DRM2 enhances plant resistance to wheat powdery mildew caused by the biotrophic pathogen *Blumeria graminis* f. sp. *tritici* in wild wheat (Geng et al., 2019). In rice, methylation of the long terminal repeat (LTR) retrotransposon “Renovator” enables the expression of the rice blast resistance gene *Pit* and confers resistance to *Magnaporthe grisea* (Hayashi et al., 2009). Similarly, hypomethylation in promoters of defence genes is associated to increased resistance in canola against *Leptosphaeria maculans,* the causal agent of blackleg disease in brassica plants (Tirnaz et al. 2020). Although studies on DNA methylation regulating immunity against post-harvest pathogens are limited, a notable example in *Citrus sinensis* (blood orange), found that high methylation levels at the promoters of the *DFR* and *RUBY* genes (both involved in anthocyanin biosynthesis) affected resistance to *Penicillium digitatum* (Sicilia et al., 2021). The above-mentioned examples, along with additional evidence highlighting the role of DNA methylation in plant responses to pathogen stress, are summarized in Additional file 1: Table S1.

## Chromatin remodelling complexes

**Fig. 3:**
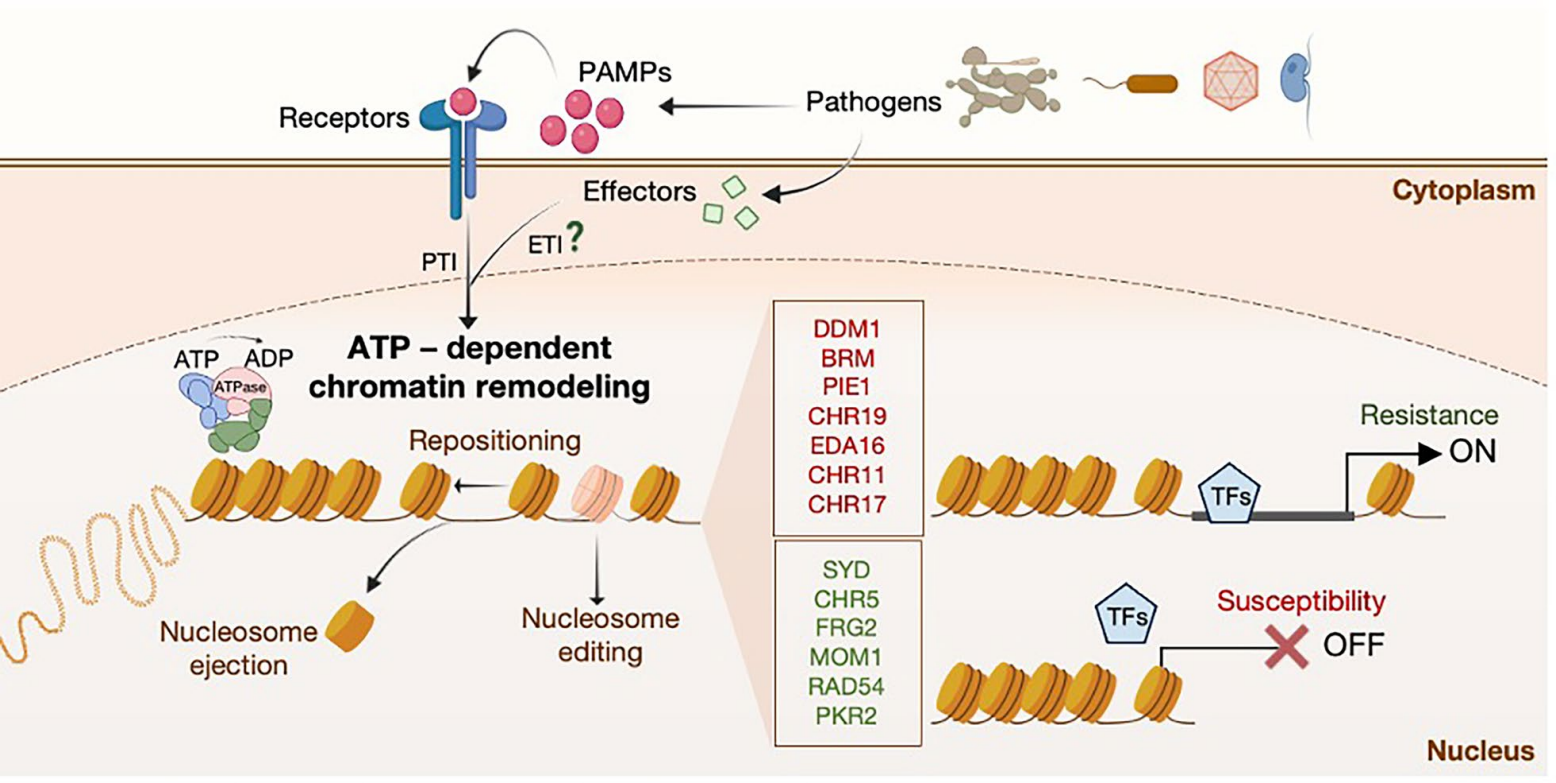
Chromatin remodelling ATPases facilitate or restrict transcriptional immune responses. Mutants of negative regulators (red) are more resistant to pathogens in comparison to WT plants and mutants of positive regulators (green) are more susceptible to pathogens. It is currently unknown which chromatin remodelling ATPases are linked to ETI activation

Histone modifications and DNA methylation involve the addition or removal of chemical tags on histone tails or DNA, whereas chromatin remodeling refers to physical alterations in nucleosome positioning. Chromatin remodelling is driven by ATP-dependent chromatin remodelling complexes (CHRs). Their basic function is to ensure proper density and spacing of nucleosomes through nucleosome assembly and organization, chromatin access and nucleosome editing by installing or removing histone variants (Fig. 3). As an outcome they regulate a plethora of genes involved in growth, development, and stress responses (Clapier et al. 2017). In the context of plant-pathogen interactions, several CHRs have emerged as crucial players in disease resistance and susceptibility (Additional file 2: Table S2).

One of the first complexes to be linked to pathogen responses is the SWR1 complex which contains Photoperiod-Independent Early Flowering 1 (*PIE1*) as its core ATPase and regulates H2A.Z deposition. Mutants of *PIE1* show constitutive expression of defence genes under normal growth conditions, as well as increased resistance to the bacterial pathogen *Pst* DC3000 (March‐Diaz et al., 2008). In contrast, Brahma (*BRM*), an ATPase of a SWI/SNF subfamily, also causes the upregulation of defence genes under normal growth conditions and has been shown to interact with a variety of histone modifications (Bezhani et al. 2007; Johnson et al. 2015).

Interestingly, some CHRs can antagonize transcriptional activators and thus repress gene expression. *BRHIS1 (BIT-responsive Histone-interacting SNF2 ATPase 1*), the rice ortholog to *A. thaliana FRG2* (also known as *CHR28*), can suppress rice innate immunity against pathogen *Magnaporthae oryzae* through binding to specific mono‐ubiquitinated histone H2A and H2B variants near the promoter regions of defence genes. The binding of *BRHIS1* obstructs their further mono‐ubiquitination and thus restricts gene expression to a basal level in healthy rice (Li et al., 2015). Additionally, the SWI2/SNF2 subfamily member Splayed (*SYD*) may repress *Suppressor of NPR1-1 Constitutive (SNC1)* transcription by antagonizing transcriptional activators (Johnson et al. 2015). *SNC1* is a well characterized intracellular immune receptor that is involved in pathogen secreted effector recognition and ETI initiation and is vital for the activation of plant resistance against bacterial pathogens (Hyun et al., 2016). A *SYD* loss‐of‐function shows enhanced resistance to the bacterial pathogen *Pst* DC3000 but increased susceptibility to *B. cinerea* (Walley et al. 2008; Johnson et al. 2015).

Other CHRs are key components in DNA methylation, like Decreased DNA Methylation (*DDM1*), that is also involved in suppressing *SNC1* expression. *A. thaliana ddm1* mutant exhibits decreased DNA methylation and the resulted transcriptional repression of plant defence genes during a pathogen attack (Li et al. 2010). Unlike *SYD* and *DDM1*, the *A. thaliana* CHR5 is a positive regulator of immune responses, which activates *SNC1* transcription and its mutant is more susceptible to bacterial pathogens. While it displayed global higher nucleosome occupancy at promoter regions near the transcription start site (TSS) which is associated with lower transcription rates, nucleosome occupancy near *SNC1* did not appear to be different between the WT and *chr5* mutant (Zou et al. 2017). CHR5 can also repress H3K27 methylation levels to regulate gene expression (Shen et al. 2015). Therefore, the transcription of *SNC1* may be co‐regulated by different CHRs through DNA methylation, histone modifications, and antagonism of transcriptional activators. This multilayered regulation was further supported by a comprehensive study by Yang et al,. which investigated the epigenetic network involved in *SNC1* activation (Yang et al. 2023).

Furthermore, multiple CHRs contribute to PTI and SAR signalling responses. Recently, it was proven that *Embryo Sac Development Arrest 16 (EDA16)*, under *Pst* DC3000 infection, selectively regulates a defined subset of genes involved in redox signalling through nucleosome repositioning (Pardal et al. 2021). Also, Kang et al., showed that several inducible genes, including key components of SA and JA pathways, were upregulated in the *chr19* mutant under normal growth conditions. However, this impaired proper JA-mediated defense, making the mutant more susceptible to the necrotrophic fungal pathogen *B. cinerea* (Kang et al. 2022). It is also worth mentioning that although there is scarce evidence that chromatin remodelling factors contribute to priming, Torre et al., provided data that strongly support the chromatin factor *Morpheus Molecule 1 (MOM1)* as an indirect but important component of priming against *Pseudomonas* sp. in *A. thaliana*, where *mom1* mutants showed an increased primary root susceptibility to the inhibitory effect of the priming inducers AZA (Azelaic acid), BABA (β-aminobutyric acid), and PIP (N-hydroxypipecolic acid) (Miranda de la Torre et al., 2023). Chromatin remodeling has also been implicated in regulating ETI-induced transcriptional responses, but it remains unclear which chromatin remodeling ATPases are specifically linked to ETI activation (Ding. et al., 2021).

These interactions highlight the complex regulatory networks that govern plant immunity. However, it is important to note that nearly all studies on CHRs have focused exclusively on leaves, leaving root immunity, a critical interface with soil-borne pathogens relatively unexplored.

## Non-coding RNAs

Noncoding RNAs (ncRNAs) are functional RNAs that are not translated into proteins and can be broadly categorized into housekeeping types (tRNAs, rRNAs, snRNAs, and snoRNAs) and regulatory types (miRNAs, siRNAs, piRNAs, and lncRNAs). Regulatory ncRNAs play crucial roles in gene expression by guiding transcript cleavage, repressing translation, or modulating chromatin through DNA and histone methylation (Morris and Mattick, 2014; Waititu et al. 2020). This section highlights key ncRNA-mediated defence mechanisms, including post-transcriptional gene silencing (PTGS), host-induced gene silencing (HIGS), and cross-kingdom RNAi.

In plant immunity, ncRNAs, such as miRNAs and siRNAs, mediate PTGS by suppressing foreign nucleic acids and fine-tuning NLR gene expression to balance defence and prevent excessive immunity during pathogen interactions. In rice, overexpression of miR398b suppressed superoxide dismutase transcripts, leading to elevated ROS levels and increased resistance to *M. oryzae* (Li et al. 2014, 2019). Similarly, in *A. thaliana*, miR400 regulates pentatricopeptide repeat genes, modulating ROS homeostasis and influencing susceptibility to *B. cinerea* (Park et al. 2014). In cereals, wheat miR408 was shown to regulate resistance against stripe rust (*Puccinia striiformis* f. sp. *tritici*) through targeting a chemocyanin-like protein gene (Feng et al. 2013), while in tomato, the downregulation of miR482f and miR5300 during *F. oxysporum* infection permitted the expression of NB-domain resistance genes in resistant cultivars (Ouyang et al. 2014). A similar mechanism is seen in cotton, where suppression of miR482 enhances expression of NBS (Nucletide binding site)-LRR genes to defend against *V. dahliae* (Zhu et al. 2013). Finally, in fruit crops, *Malus domestica* resistance to *Diplocarpon mali* was linked to reduced expression of Md-miRLn11, thereby allowing higher accumulation of an NBS-LRR resistance protein (Ma et al. 2014).

Next, HIGS has proved the potential of engineering plants producing silencing RNAs against invading pathogens, making it a powerful applied strategy for crop protection (Nowara et al., 2010; Pliego et al., 2013). HIGS has been successfully demonstrated in cotton, where *V. dahliae* infection induces miR159 and miR166, which directly target fungal virulence-related genes to suppress invasion (Zhang et al. 2016). This mechanism is not limited to cotton and *V. dahliae* as it was also observed across a range of pathogens, including *B. graminis*, *Fusarium verticillioides* and the oomycetes *Bremia lactucae*, *P. infestans*, and *Phytophthora capsici* (Koch et al., 2013; Govindarajulu et al., 2015; Jahan et al., 2015; Hou et al., 2019). In contrast to HIGS, cross-kingdom RNAi occurs naturally when siRNAs move bidirectionally between plants and pathogens. *A. thaliana* secretes exosome-like vesicles containing siRNAs into *B. cinerea*, where they silence genes critical for pathogenicity (Cai et a., 2018). Similarly, tomato miR1001 suppresses *B. cinerea* growth and virulence by targeting metallopeptidase and endopeptidase genes (Meng et al. 2020). Conversely, *B. cinerea* delivers its own siRNAs into host cells to silence resistance genes, a process dependent on fungal Dicer-like proteins Bc-DCL1 and Bc-DCL2 (Wang et al. 2016). While cross-kingdom RNAi has been extensively documented in plant-fungus and plant–insect interactions, evidence for similar mechanisms for bacterial pathogens, which lack conventional RNAi machinery, remains scarce. A promising study by Singla-Rastogi showed that *A. thaliana* transgenic plants expressing siRNAs that target virulence factors of a *P. syringae* strain, reduces its pathogenesis (Singla-Rastogi et al., 2019).

## Conclusions and translation to crop protection

From the studies highlighted in this review, it is evident that there is a high level of conservation in the roles of key modulators of DNA methylation, histone modifications, and chromatin remodeling across plant species. DNA methylation and histone modifications consistently regulate defense gene expression, indicating that these conserved mechanisms can be leveraged to enhance crop disease resistance. Additionally, these epigenetic layers are interconnected, with DNA methylation influencing histone modification patterns and chromatin accessibility, creating a coordinated network that fine-tunes plant immunity.

Building on this understanding of conserved epigenetic regulation, advanced sequencing and epigenomic profiling methods offer powerful tools to reveal novel epigenetic marks that may contribute to plant immunity. For instance, ChIP-seq (Chromatin immunoprecipitation sequencing), ATAC-seq (Assay for transposase-accessible chromatin sequencing), and MNase-seq (Micrococcal nuclease sequencing) collectively provide complementary insights into chromatin dynamics, including histone modifications, accessible regulatory regions, and nucleosome positioning during plant stress responses. More recently, high-throughput methods such as CUT&RUN (Cleavage under targets and release using nuclease) and CUT&TAG (Cleavage under targets and tagmentation) have gained popularity for their efficiency and reduced input requirements, enabling precise mapping of histone modifications and protein-DNA interactions even in limited or heterogeneous plant samples.

While these bulk methods have proven valuable for capturing general epigenetic landscapes at the tissue level, they often mask the heterogeneity of responses between individual cells. Emerging single-cell approaches, including single-cell bisulfite sequencing (scBS-seq) for profiling DNA methylation patterns and single-cell ATAC-seq (scATAC-seq) for identifying chromatin accessibility at the cellular level, are beginning to fill this gap. Although such techniques remain underutilized in plant immunity research, they hold enormous promise for uncovering cell-type-specific epigenetic responses to pathogens. Notably, work in plant developmental biology has already demonstrated the transformative potential of single-cell epigenomics (Zhan et al. 2023; Dorrity et al. 2021). Extending these approaches to plant-pathogen interactions represents a critical frontier, offering a foundation for context-specific epigenome editing strategies that minimize the risks of off-target effects on growth and development.

Beyond discovery tools, practical applications of epigenetic regulation are now being explored with the goal of improving crop resilience to pathogens. Among various epigenetic modifications, DNA methylation stands out due to its relatively high heritability across generations (Quadrana and Colot, 2016). This stability makes it an attractive target for engineering disease resistance. Advances on targeted epigenome engineering methods such as artificial zinc finger proteins (ZF) and CRISPR/dCas9 systems have already been developed to deliver DNA methylation or demethylation enzymes to specific genomic regions (Gallego-Bartolome., 2020; Gardiner et al., 2022). For example, in cassava, a ZF-based tool successfully methylated a promoter targeted by *Xanthomonas phaseaoli* pv. *manihotis* (*Xam*), the causal agent of cassava bacterial blight, preventing its activation and increasing resistance (Veley et al. 2023). Despite promising early successes, several key challenges remain in translating epigenetic insights into practical tools for crop improvement. To facilitate this transition, we propose a set of guiding principles to inform the strategic selection and application of epigenetic approaches aimed at enhancing crop resistance to pathogens:

## Guiding principles for applying epigenetics in crop disease resistance

1. *Prioritize epigenetic marks involved in PTI and ETI with defined immune functions*
  Focus on epigenetic modifications that have well-characterized roles in PTI and ETI, particularly those that directly regulate the expression of immune-related genes.
2. *Leverage knowledge from effectoromics*
  Utilize epigenetic marks targeted by pathogen effectors in a direct or indirect manner though histone-DNA interactions, as these represent critical control points in host-pathogen interactions and offer strategic leverage for enhancing resistance.
3. *Focus on organ- or cell-type-specific epigenetic regulation*
  Target epigenetic marks with spatially restricted function to reduce the risk of pleiotropic effects and maintain overall plant performance, ensuring that disease resistance is enhanced without compromising growth, development or crop yield.
4. *Exclude marks that can lead to misregulation of immunity*
  Avoid epigenetic modifications that alter the expression of PRRs and NLRs or disrupt the hormonal homeostasis of the plant, to avoid autoactivation of immunity and pleiotropic effects.
5. *Consider environmental stability of epigenetic marks*
  Prioritize modifications that remain robust across varying field conditions and exclude those highly susceptible to changes of temperature, light, and abiotic stress.

## Supplementary Information

**Additional file 1: Table S1.** Involvement of DNA methylation in the response of plants toinfections caused by pathogenic fungi, oomycetes, and bacteria.

**Additional file 2: Table S2. **Role of ATP-dependent chromatin remodelling factors in plantimmune responses.

## Data Availability

Not applicable.

## Abbreviations

CHRs: Chromatin remodellers
DAMPs: Damage associated molecular patterns
ET: Ethylene
ETI: Effector triggered immunity
ETS: Effector triggered susceptibility
HATs: Histone acetyltransferases
HDACs: Histone deacetylases
HDMs: Histone demethylases
HMTs: Histone methyltransferases
IR: Induced resistance
ISR: Induced systemic resistance
JA: Jasmonic acid
MAPK: Mitogen activated protein kinase
MAMPs: Microbe associated molecular patterns
NLRs: Nucleotide binding and leucine rich‐repeat
PAMPs: Pathogen associated molecular patterns
PRRs: Pattern recognition receptors
PTI: PAMP triggered immunity
ROS: Reactive oxygen species
SA: Salicylic acid
SAR: Systemic acquired resistance
