# Supplementary material for: How to improve crop pathogen resistance with epigenetics
Source: Phytopathol Res. 2026 Jan 27;8(1):5. doi: 10.1186/s42483-025-00393-7 (PMC12835110; doi:10.1186/s42483-025-00393-7)
Supplement: Supplementary file 1 — Additional file 1: Table S1. Involvement of DNA methylation in the response of plants toinfections caused by pathogenic fungi, oomycetes, and bacteria. [file 42483_2025_393_MOESM1_ESM.docx]

**Table S1. Involvement of DNA methylation in the response of plants to infections caused by pathogenic fungi, oomycetes, and bacteria.**

| **Pathogen** | **DNA methylation mechanism** | **Plant/pathogen** | **Immunity phenotype** | **References** |
| --- | --- | --- | --- | --- |
| **Fungi** | RdDM pathway positively regulates resistance against necrotrophic fungi presumably by promoting JA signalling | *A. thaliana / B. cinerea* | Resistant | Lopez et al.,2011 |
|  | ROS1, DML2, and DML3 positively regulate expression of stress response genes | *A. thaliana / F. oxysporum* | Resistant | Le et al.,2014 |
|  | Hypomethylation of the LTR region in the promoter of the rice blast resistance gene *Pit* | *Oryza sativa / Magnaporthe grisea* | Resistant | Hayashi et al.,2009 |
|  | Silencing of MET1 (MET1 maintains the CG methylation of resistance genes) | *Morus notabilis / B. cinerea* | Resistant | Xin et al., 2021 |
|  | Hypermethylation in promoters of defence genes | *Brassica napus /*  *Leptosphaeria maculans* | Resistant | Tirnaz et al., 2020 |
|  | Downregulation of DME leads to increased susceptibility to fungal pathogens | *A. thaliana /V. dahliae* | Susceptible | Zeng et al., 2021 |
|  | Hypermethylation of transposable elements regions | *Oryza sativa / M. oryzae* | Susceptible | Cui et al., 2021 |
|  | Differential methylation of a set of R genes | Melon *(Cucumis melo* L.*) / Podosphaera xanthii* | Resistant | Wang et al., 2021 |
|  | Hypermethylation at the DFR and RUBY promoters (genes involved in the anthocyanin biosynthetic pathway) | Blood orange *(Citrus sinensis* L*. (Osbeck)) /*  *Penicillium digitatum* | Resistant | Sicilia et al., 2021 |
|  | Differentially methylated regions with CHH-hypomethylated | *Aegilops tauschii /*  *Blumeria graminis* f. sp. *tritici (Bgt)* | Resistant | Geng et al., 2019 |
|  | Hypomethylation in *ago4* suppresses of JA -dependent defence | 1. *thaliana/ B. cinerea* | Susceptible | Lopez et al., 2011 |
|  | Hypomethylation in *cmt3* enhances SA-dependent response | 1. *thaliana/ Hpa* | Resistant | Lopez Sanchez et al., 2016 |
|  | Hypomethylation in *met1* enhances SA-dependent response | 1. *thaliana/ Pst & Pst* | Resistant | Yu et al., 2013 |
|  | Hypomethylation in *ros1* enhances JA-dependent response | 1. *thaliana/ Plectosphaerella cucumerina* | Resistant | Lopez Sanchez et al., 2016 |
|  | Hypomethylation in *rdr6* loses transfer siRNAs that target pathogen genes | 1. *thaliana/ Bc* | Susceptible | Cai et al., 2018 |
|  | Hypomethylation in *nrpe1* suppresses JA -dependent response | 1. *thaliana/ Pc and Bc* | Susceptible | Lopez et al., 2011; Lopez Sanchez et al., 2016 |
|  | Hypomethylation in *nrpd2* suppresses JA-dependent response | 1. *thaliana/ Pc and Bc* | Susceptible | Lopez et al., 2011 |
|  | Hypomethylation in *dcl2/3/4* leads to transfer failure of siRNAs that target pathogen related genes | 1. *thaliana/ Bc* | Susceptible | Cai et al., 2018 |
|  | Hypomethylation in *drd1* suppresses JA-dependent response | 1. *thaliana/ Pc* | Susceptible | Lopez et al., 2011 |
|  | Hypomethylation in *rdr2* suppresses JA-dependent response | 1. *thalina/ Pc* | Susceptible | Lopez et al., 2011 |
|  | Hypomethylation in *drm1/drm2* suppress JA -dependent response | 1. *thaliana / Pc* | Susceptible | Yu et al., 2013 |
|  | Hypomethylation in *rdr1* enhances SA-dependent response | 1. *thaliana/ P. cucumerina* |  | Lopez et al., 2011 |
| **Oomycetes** | Hypomethylation in *cmt3, drd1,* and *nrpe1* enhance SA-dependent response | 1. *thaliana/ Hyalonospora arabidopsis (Hpa)* | Resistant | Lopez Sanchez et al., 2016 |
| **Bacteria** | Hypermethylation in *ros1* at the promoters of *RMG1* and *RLP43* | 1. *thaliana/ Pst* | Susceptible | Yu et al., 2013; Halter et al., 2021 |
|  | Hypomethylation in *ago4* promotes RDR2 and DCL3 independent susceptibility | 1. *thaliana/ Pst* | Susceptible | Agorio and Vera, 2007 |
|  | Hypomethylation in *drd1* enhances SA-dependent response | 1. *thaliana/ Pst* | Resistant | Dowen et al., 2012;  Lopez Sanchez et al., 2016 |
|  | Hypomethylation in *rdr2* enhances SA-dependent response | 1. *thaliana/ Pst* | Resistant | Dowen et al., 2012 |
|  | Hypomethylation in *rdr6* exhibits RPS2-mediated ETI | 1. *thaliana/ Pst* | Resistant | Katiyar-Agarwal et al., 2006 |
|  | Hypomethylation in rdr6 (unknown defence response) | 1. *thaliana/ Pst* | Resistant | Dowen et al., 2012 |
|  | Hypomethylation in *nrpe1*enhances SA-dependent response | 1. *thaliana / Pst* | Resistant | Lopez et al., 2011 |
|  | Hypomethylation in *nrpd2* enhances SA-dependent response | 1. *thaliana /Pst* | Resistant | Lopez et al., 2011; Yu et al., 2013 |
|  | Hypomethylation in *nrpd1/nrpe1*enhance SA- dependent response | 1. *thaliana /Pst* | Resistant | Lopez et al., 2011 |
|  | Mutants *drm1*/*drm2* exhibit primed state of defences response | 1. *thaliana /Pst* | Resistant | Cambiagno et al., 2021 |
|  | Hypomethylation in *drm1/drm2/cmt3 (ddc)* enhance the SA-dependent response | 1. *thaliana /Pst* | Resistant | Dowen et al., 2012; Luna et al., 2012; Yu et al., 2013; Cambiagno et al., 2021 |
|  | Hypomethylation in *dcl2/3/4* (unknown defence response) | 1. *thaliana/ Pst* | Resistant | Dowen et al., 2012 |
