# Supplementary material for: How to improve crop pathogen resistance with epigenetics
Source: Phytopathol Res. 2026 Jan 27;8(1):5. doi: 10.1186/s42483-025-00393-7 (PMC12835110; doi:10.1186/s42483-025-00393-7)
Supplement: Supplementary file 2 — Additional file 2: Table S2. Role of ATP-dependent chromatin remodelling factors in plantimmune responses. [file 42483_2025_393_MOESM2_ESM.docx]

**Table S2. Role of ATP-dependent chromatin remodelling factors in plant immune responses.**

| **Protein family** | **Gene** | | **Influence on immunity** | **References** |
| --- | --- | --- | --- | --- |
| Snf1 | AT5G66750 | *CHR1/DDM1* | Positive | Li et al., 2010 |
|  | AT2G02090 | *CHR19/ ETL1* | Positive | Kang et al., 2022 |
|  | AT3G12810 | *CHR13/PIE1* | Positive | March‐Diaz et al.,2008 |
| Snf2 | AT2G28290 | *CHR3/SYD* | Negative | Walley et al., 2008; Johnson et al., 2015 |
|  | AT2G46020 | *CHR2/ BRM* | Positive |  |
|  | AT4G31900 | *CHR7/PKR2* | Positive | Pardal et al.,2021 |
|  | AT2G13370 | *CHR5* | Negative | Zou et al., 2017 |
| ISWI | AT3G06400 | *CHR11* | Positive | Liu et al., 2021 |
|  | AT5G18620 | *CHR17* | Positive | Liu et al., 2021 |
| Rad5 | AT1G08060 | *MOM1* | Negative | Miranda de la Torre et al., 2023 |
| Rad54 | AT3G19210 | *CHR25/ RAD54* | Positive | Pardal et al., 2021 |
| Ris-1 | AT1G50410 | *CHR28/FRG2* | Negative | Li et al., 2015 |
|  | AT1G61140 | *EDA16/FRG4* | Positive | Pardal et al., 2021 |
